# EnrichKit: a multi-omics tool for livestock research

**DOI:** 10.3389/fgene.2025.1573374

**Published:** 2025-05-13

**Authors:** Lihe Liu, Francisco Peñagaricano

**Affiliations:** Department of Animal and Dairy Sciences, University of Wisconsin-Madison, Madison, WI, United States

**Keywords:** software, overrepresentation analysis, P-value aggregation, RNA-seq, WGBS-seq

## Abstract

The increasing applications of omics technologies in livestock research highlights the need for tools capable of interpreting preliminary signals, such as mapping genomic coordinates to gene features and annotating gene lists for functional characterization. These tools should effectively leverage various biological databases for comprehensive analysis. Additionally, the development of user-friendly interfaces is essential to broaden the accessibility and enable a wider range of users to contribute more effectively to the field of livestock genomics. EnrichKit provides friendly graphical user interface and superior efficiency in data management and computational analysis by integrating various public databases and statistical algorithms. Its functionalities are showcased through applications in DNA methylation analysis, gene co-expression network analysis, and differential gene expression analysis. The comparative analysis with existing tools underscores EnrichKit advantages in terms of species-specific gene-set libraries and user accessibility. EnrichKit significantly advances the interpretation of omics studies in livestock genomics. Its tailored approach for species-specific analysis, combined with a comprehensive computational framework, positions it as a valuable tool for researchers. The potential of EnrichKit to transform livestock genomics research is evident, opening avenues for future enhancements and broader applications in the livestock omics research field.

## 1 Introduction

There has been a remarkable development of omics technologies, and it has paved the way for advancements in the era of systems biology. For example, genomics provides a comprehensive understanding of an organism’s genetic blueprint, including disease susceptibility, evolutionary history, trait inheritance, and interactions with environmental factors. Transcriptomics measures the expression level of transcripts in a specific cell and in a particular state, enabling insights into active biological pathways, disease-associated genes, environmental responses, and cellular regulatory networks. There are growing examples demonstrating such omics applications in livestock production and health traits studies, such as annotating livestock animal genomes (Andersson et al., 2015; Tuggle et al., 2016), identifying genetic variants (Abdollahi-Arpanahi et al., 2021; Xiang et al., 2021), deciphering complex processes (Liu et al., 2020; Liu et al., 2021), unraveling intricate molecular mechanisms (Canovas et al., 2014) and improving breeding practices (Porto Neto et al., 2011; Pacheco et al., 2020). In addition, the multi-omics approach combines data from various omics types. By integrating these datasets, researchers gain a holistic view of biological systems, revealing intricate interactions and networks. This comprehensive perspective offers deeper insights than single-omics studies, aiming for a systems-level understanding of biology.

In biological studies using omics-driven approaches, the primary results are often characterized by genomic coordinates-based signals and gene-list based signals. For instance, Genome-Wide Association Studies (GWAS) can yield loci and genetic markers associated with phenotypic traits through the identification of single nucleotide polymorphisms (SNPs) within the genomic coordinates. Similarly, Whole-Genome Bisulfite Sequencing (WGBS) generates comprehensive maps of cytosine methylation patterns across the genome coordinates, e.g., differentially methylated sites and regions, which are crucial for understanding the epigenetic changes associated with the traits of interest. Further, RNA Sequencing (RNA-Seq) can provide gene expression profiles, where the transcriptional activity of genes is measured, generating lists of candidate genes under various conditions or treatments, e.g., differential expression (Louvandini et al., 2020) or differential exon usage (Liu et al., 2021). These preliminary outputs need additional analyses to uncover the underlying biological mechanisms. Specifically, signals based on coordinates must be associated with proximal gene features to gain meaningful biological insights. Moreover, gene lists typically require functional characterization through the enrichment analysis of gene sets.

A vast array of public databases and statistical algorithms have emerged and can be integrated to tackle the complexities of downstream biological interpretation of the preliminary omics outputs. Multiple entities actively provide genome annotations in GTF format, such as Ensembl (Martin et al., 2023), *NCBI* (Sayers et al., 2023), UCSC Genome Browser (Lee et al., 2022), and more. These annotations delineate genomic features along with their corresponding coordinates, which can be utilized to associate expression levels or genetic variations with specific genomic elements. Various sources compile gene sets of differing nature, for example, functional categories such as Gene Ontology (GO) (Ashburner et al., 2000) and Interpro (Mitchell et al., 2019), pathway categories like *KEGG* (Kanehisa and Goto, 2000) and Reactome (Jassal et al., 2020), and comprehensive categories Medical Subject Headings (*MeSH*) (Nelson et al., 2004), among others. Such gene sets are fundamental inputs for downstream enrichment analysis. In addition, several analytic algorithms have been developed to decipher the biological significance of candidate gene lists. Overrepresentation analysis (ORA) was proposed as the first-generation method and is commonly used to examine whether predefined gene sets are represented more than expected by chance, while Gene Set Enrichment Analysis (GSEA) is an advanced approach that assesses gene set enrichment across an entire sorted candidate gene list (Subramanian et al., 2005). The integration of these databases and tools has profoundly enhanced our understanding of molecular biology.

While several software tools have been developed for these analyses, there is a notable gap in the availability of tools explicitly tailored to primary livestock species for comprehensive multi-omics interpretation analysis, i.e., both mapping coordinates to annotations and gene-set enrichment analysis. Moreover, similar existing tools often require a degree of programming expertise and lack a user-friendly graphical interface that would enable researchers without coding skills to perform these analyses efficiently. In this work, we present a novel software, EnrichKit, a web-based application designed to facilitate the biological interpretation of coordinate-based and gene-list-based signals in major livestock species. The software features an intuitive graphical interface that eliminates the need for coding, making complex analyses accessible to a broader range of scientists. Additionally, the asynchronous processing capabilities and solid computational framework enable expedient handling of extensive multi-omics datasets.

## 2 Methods

### 2.1 Overview of EnrichKit

**FIGURE 1:**
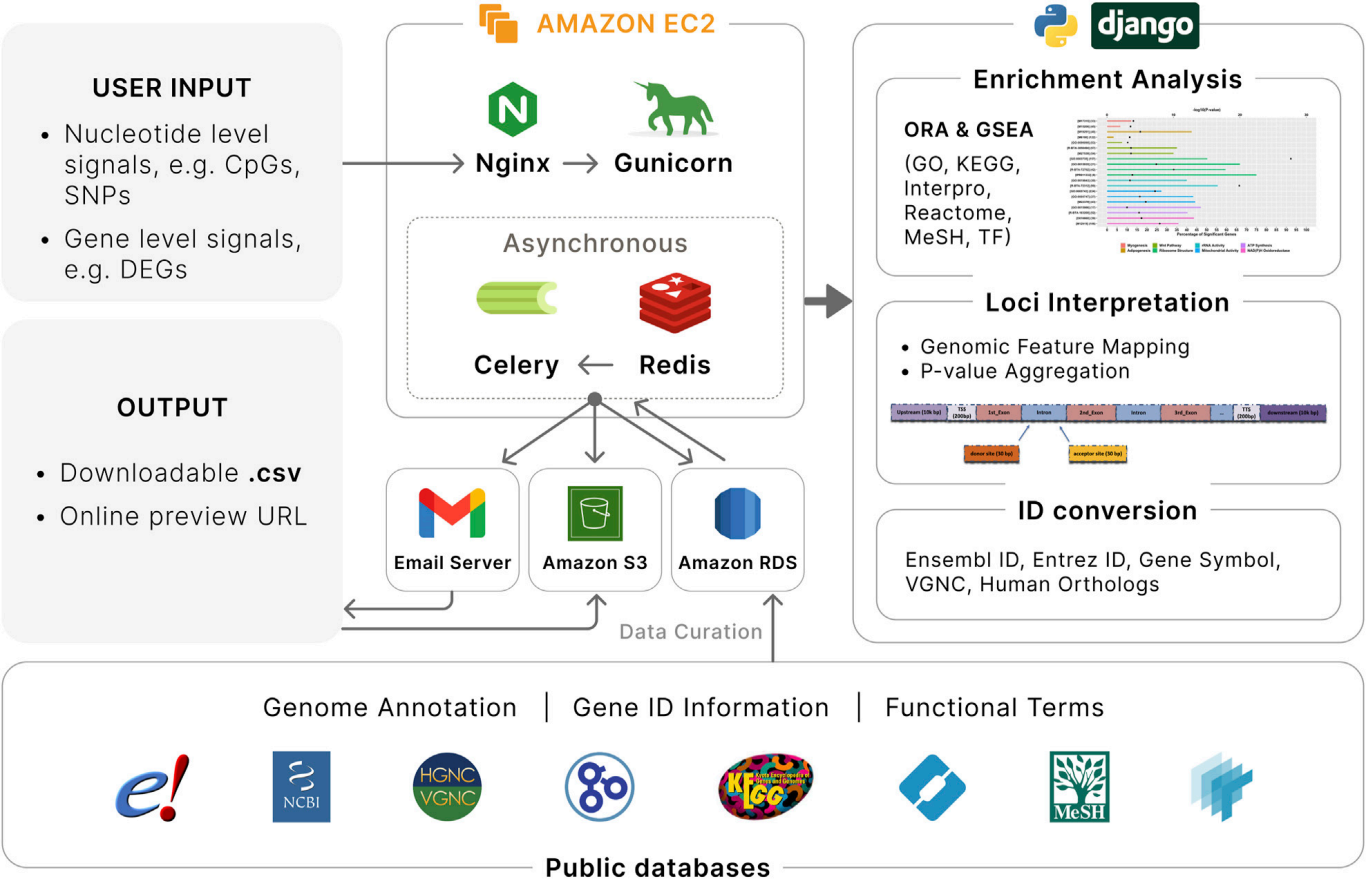
Overview of EnrichKit web application. Various public databases are used to retrieve relevant data. User input to EnrichKit includes either nucleotide level signals or gene level signals. Two type of gene-set enrichment analyses are available to perform functional characterization of input gene lists. Genomic feature mapping and P-value aggregation are available to associate nucleotide level signals with adjacent genes. Gene ID conversion provides correspondence among different gene identifiers among different sources and human orthologs. The application runs on Amazon Web Services with efficient workflow.

We developed EnrichKit, a web-based application for biological interpretation of user-supplied lists of coordinate-based and gene-list-based signals in livestock species (Figure 1). This application is implemented using a Python/Django framework with *MySQL* database integration. It is publicly available online (http://enrichkit.info/). The source code for the web application is available via GitHub (https://github.com/liulihe954/EnrichKitWeb). In addition, to facilitate analysis involving larger input file and provide alternatives during webapp outages, we developed a set of supplementary python scripts that replicate the webapp’s functions locally on user devices. The python scripts and instructions can be found via GitHub (https://github.com/liulihe954/EnrichKitPy).

In this version, there are six functions provided: gene ID conversion, mapping of genomic coordinates, P-value aggregation, overrepresentation analysis, gene set enrichment analysis, and transcription factor enrichment analysis. The current version supports six major livestock organisms, including cow (Bos taurus), goat (Capra hircus), sheep (Ovis aries), chicken (Gallus gallus), pig (Sus scrofa) and horse (Equus caballus), which are the major livestock species where omics technologies have been extensively applied to (Thomann et al., 2023). For each species, EnrichKit integrates all genomics features from the Ensembl genome annotation GTF file and relevant gene sets from five popular libraries, including Gene Ontology (GO), Interpro, *KEGG*, Reactome and Medical Subject Headings (*MeSH*). Specifically, species-specific gene sets are retrieved from each library, ensuring the preservation of most biological information that is unique to each species.

### 2.2 Software architecture and asynchronous workflow

Our web application is hosted on Amazon Web Services (AWS) Elastic Compute (EC2) cloud, providing a robust and scalable cloud environment. The backend database is managed through AWS RDS *MySQL* instance, ensuring efficient data management and storage. For handling file storage, e.g., storing user input and analysis output, we utilize AWS S3 buckets, which offer secure and scalable storage solutions. The backend architecture is further strengthened by using Nginx as a reverse proxy, which efficiently manages client requests and server responses. We employ Gunicorn as our Python Web Server Gateway Interface (WSGI) HTTP server, enhancing our application’s ability to handle concurrent requests with improved performance. Asynchronous tasks queue and background processes are implemented to enhance user experience, e.g., eliminate the need for users to wait for an analysis to complete and reduce the risk of disconnections during long-running operations. Specifically, we integrate the Django Celery worker queue with a Redis database to ensure smooth handling of background processes in a first-in-first-out style. Email notifications have been implemented to inform users of input submissions and provide updates on the status of their analysis. All these components and processes are effectively managed and monitored using Supervisor, which aids in maintaining the application’s stability and performance. Each job submission is assigned a universally unique identifier (UUID), which can be used to freely retrieve the results within a week.

### 2.3 Public resources and data integration

EnrichKit employs a broad spectrum of publicly available data to construct a comprehensive internal database, enabling the supported functions to make inquiries effectively. Genome annotation GTF files were downloaded from Ensembl FTP portal, Gene ID information and correspondence were retrieved from various sources, including *NCBI*, Vertebrate Gene Nomenclature Committee (*VGNC*) (Jones et al., 2023) and HUGO Gene Nomenclature Committee (*HGNC*) (Seal et al., 2023). For functional categories and biological pathway gene sets, Ensembl Biomart service RESTful API were used to retrieve Gene Ontology and Interpro gene sets, various *KEGG* API were used to retrieve *KEGG* gene sets, Reactome data portal was used to retrieve reactions gene sets and Bioconductor AnnotationHub was used to retrieve *MeSH* term (category D and G) gene sets. Table 1 shows a description of the data sources. Python scripts were developed to clean, parse, and format these raw data to generate tables for populating a relational database. All scripts used for this purpose can be found via GitHub (https://github.com/liulihe954/EnrichKitDB) and the generated portable SQLite database object is available via Zenodo (https://doi.org/10.5281/zenodo.10257551). These tables were loaded into *MySQL* database which was later integrated into the web application program using the Django ORM framework. Table 2 shows the descriptive statistics of the data items in the database.

**TABLE 1:** Description of data sources.

Data source	Data type	URL
Ensembl genome database	Genome annotation	https://useast.ensembl.org/info/data/ftp/index.html
NCBI gene2ensembl	Gene ID mapping between NCBI (Entrez ID) and Ensembl ID	http://ftp.ncbi.nih.gov/gene/DATA/gene2ensembl.gz
NCBI gene_info	Various gene ID information	http://ftp.ncbi.nih.gov/gene/DATA/gene_info.gz
VGNC	Vertebrate gene symbols and names	http://ftp.ebi.ac.uk/pub/databases/genenames/vgnc/tsv/all/locus_groups/all_protein-coding_gene_All.txt
HGNC	Human gene symbols and names	https://storage.googleapis.com/public-download-files/hgnc/tsv/tsv/locus_groups/protein-coding_gene.txt
Biomart service	Gene ID, GO terms, Interpro terms	https://useast.ensembl.org/info/data/biomart/index.html
KEGG API	KEGG pathways	https://www.kegg.jp/kegg/rest/keggapi.html
Reactome	Reactome pathways	https://reactome.org/download/current/NCBI2Reactome_All_Levels.txt
AnnotationHub	MeSH terms	https://bioconductor.org/packages/release/bioc/html/AnnotationHub.html
tftargets R package	Human transcription factor-target relationship	https://github.com/slowkow/tftargets

**TABLE 2:** Description of Database records.

Species	Genes	Canonical feature^a^	Computed feature^b^	GO term	KEGG pathway	Interpro term	Reactome term	MeSH term
*Bos taurus*	35,606	786,245	791,619	15,555	363	17,795	1,729	10,558
*Capra hircus*	25,107	520,284	588,155	15,656	363	17,383	N/A	N/A
*Ovis aries*	30,584	734,201	738,446	7,243	363	15,072	N/A	N/A
*Gallus gallus*	29,521	717,853	732,152	15,109	192	16,136	1,604	10,558
*Sus scrofa*	34,270	694,249	687,726	16,393	363	12,487	N/A	10,558
*Equus caballus*	37,754	1,003,499	946,671	16,134	363	17,413	1,622	10,558
Total	192,842	4,456,331	4,484,769	86,090	2,007	96,286	4,955	42,232

^a^ Canonical Features: coding sequence (CDS), exon, five prime UTR, start_codon, stop_codon, three prime UTR, transcript.

^b^ Computed Features: downstream, intron, splice acceptor, splice donor, transcription starting site (TSS), upstream.

### 2.4 Mapping of genomic coordinates

The genomic coordinates mapping function takes specific genomic locations and cross-references them with an annotated genome to ascertain associated gene features. The output details the overlapping of genomic features, which provides critical insights into the functional implications of those coordinates. Specifically, there are 12 type of genomic features encoded to match the input coordinates, including upstream, downstream, exons, introns, start codon, stop codon, coding sequence (CDS), 5′UTR, 3′UTR, transcript, splice donor (5′end of the intron), splice acceptor (3′end of the intron). Additionally, users can customize the parameters to specify the length of upstream and downstream regions, as well as splice donors and splice acceptors, to suit the study requirements.

### 2.5 P-value aggregation

Local P-value aggregation enables the transition from fine-grained, site-specific preliminary measurements, such as raw P-values, to biologically interpretable and statistically robust gene-level summaries before performing genome-wide multiple testing corrections. This approach can potentially address heterogenous effects across a gene, reduces the multiple testing burden, mitigates signal sparsity, aligns results with gene-level functional annotations, and facilitates the integration of multi-modal data. Our approach follows a two-step process. Initially, this function accepts a list of genomic coordinates and maps each coordinate to its nearest gene using an annotation reference. Subsequently, for each gene, it aggregates the P-values associated with the previously mapped genomic coordinates (each coordinate contributes a signal input) using a predefined statistical aggregation method. The output is an aggregated P-value for each gene, providing a collective significance that reflects the combined evidence from all signals mapped to a certain gene. Currently, four aggregation methods are implemented, including Fisher’s combination test, Sidak’s combination test, Simes’ combination test, and the FDR method. Each method represents a unique strategy for aggregating raw P-values, reflecting different statistical philosophies and assumptions.

### 2.6 Overrepresentation analysis (ORA) and gene set enrichment analysis (GSEA)

ORA identifies statistically significant overrepresentation of a predefined gene set, such as biological functions or pathways, within a subset of “interesting” genes as compared to a background gene list. This function employs the hypergeometric test to determine if certain genes occur in a set more frequently than expected by chance, thereby providing insights into the underlying biological processes associated with the gene list of interest. GSEA is a ranking-based approach that determines if a predefined gene set shows statistically significant, concordant differences between two biological states. It employs the Kolmogorov-Smirnov test to evaluate the distribution of genes across a ranked list. It provides an enrichment score that reflects whether members of the provided gene set are randomly distributed or primarily found at the top or bottom, indicating potential association with the biological states of interest. Overall, a total of 218,902 pathways from five distinct databases are available for ORA and GSEA, distributed among six species.

### 2.7 Transcription factor enrichment analysis

A special collection of human transcription factors and target gene relationship gene sets were extracted from R package tftargets (https://github.com/slowkow/tftargets) and integrated into EnrichKit to assess the involvement of regulatory elements for the input gene list given certain biological contexts. This test employs a hypergeometric test and allows user to identify key transcription factors that may be driving the observed alterations, e.g., gene expression, in the given gene list. Such insights are vital for understanding the regulatory mechanisms underpinning gene expression dynamics in various biological states or conditions.

## 3 Results

### 3.1 Graphical user interface

EnrichKit features a dual-panel graphical user interface, optimized for both ease of use and efficient data analysis. The left panel serves as a functional directory, where users can select from a list of tools. Choosing a function triggers the right panel to display corresponding steps and options. This interface guides users through a four-step analysis process: selecting a species from a dropdown menu, entering custom parameters for the chosen function, uploading or pasting a dataset, and then providing an email address for communication and result notifications. The custom parameters collection step has a distinct design for each function. Specifically, the genomic coordinate annotation and aggregation functions allow users to select features pertinent to their research via checkboxes. Additionally, for features that permit customization, users can define and input their desired lengths in the input field. This design facilitates a flexible and tailored approach to data analysis, meeting diverse research specifications. Similarly, the custom parameters collection section for the enrichment analysis, i.e., functions for ORA and GSEA, allow users to customize their analysis by choosing from a list of available pathway libraries to be explored in the analysis.

### 3.2 Applications

#### 3.2.1 Application 1: mapping DNA methylation to genetic features

In a study aimed to investigate the impact of maternal nutrition on the epigenome of beef calves (Liu et al., 2021), EnrichKit was used to annotate each DNA methylation signal, i.e., CpG sites, across the whole bovine genome. This process was pivotal in establishing a framework for the downstream association analyses between epigenetic marks and transcriptomic variables. Specifically, EnrichKit effectively mapped 5,136,556 CpG sites in the whole bovine genome. This comprehensive mapping allowed for the quantification of methylation levels within these genomic regions, and subsequently, these metrics were used to perform association analyses between DNA methylation profiles and differential exon usage patterns. The conversion of coordinate-based signals to region-based signals using EnrichKit enabled a comprehensive understanding of the mechanisms by which DNA methylation influences alternative splicing.

#### 3.2.2 Application 2: overrepresentation analysis of differentially co-expressed genes

EnrichKit was utilized to conduct an overrepresentation analysis, pinpointing biological pathways linked to differentially connected genes (Liu et al., 2020). Utilizing RNA-Seq data and differential network analysis, we identified six co-expression networks that exhibited significant changes in gene connectivity between control and methionine-rich maternal diets. To decipher the functional implications of these disrupted networks, we used EnrichKit to search for significant functional terms among five libraries, revealing a diverse spectrum of physiological and metabolic pathways altered by the diet, including myogenesis, adipogenesis, fibrogenesis, and the canonical Wnt/β-catenin pathway. With the help of EnrichKit, this study provides vital evidence on how maternal diet variations can profoundly affect gene co-expression patterns in offspring, which may have significant implications for their developmental trajectory.

#### 3.2.3 Application 3: gene set enrichment analysis of differentially expressed genes

To demonstrate the capabilities of EnrichKit in performing Gene Set Enrichment Analysis (GSEA), we evaluated differentially expressed genes (DEGs) derived from a time-series RNA-Seq study assessing the effects of heat stress in dairy cows. Utilizing EnrichKit, we identified significant pathways that provided diverse biological insights. The functional characterization of these pathways revealed a subset of genes with altered expression profiles that are integrally involved in processes pertinent to mammary gland physiology. Notably, these processes include the extracellular matrix reorganization, epithelial cell signaling, metabolism regulation, heat shock protein response, and lipid as well as fatty acid transport, each of which is essential for mammary gland development and function.

## 4 Discussion

We evaluated the similarities between EnrichKit and other web-based enrichment analysis tools, including ShinyGO (Ge et al., 2020), Enrichr (Kuleshov et al., 2016), *g:Profiler* (Raudvere et al., 2019), MAGNET (Chen et al., 2023), and WebGestalt 2019 (Liao et al., 2019). These tools were selected because each one offer at least one function that overlaps with the functions implemented in EnrichKit. In cases where a tool has multiple implementations, e.g., API, R package integration, only the web services aspect was considered for this comparison. Here we present a summary highlighting the key features and considerations that we used in the comparison, such as, support for major livestock species, the range of functions provided, availability of livestock-specific pathway libraries, the ability to use customized background gene lists, the display of overlapping significant genes, access to an internal database and its reproducibility, code availability, and the implementation of asynchronous workflows. Table 3 summarizes this comparison between EnrichKit and five other available tools.

**TABLE 3:** Comparisons to other similar web-based tools.

Name	Supporting major livestock species	Accessible functions	Livestock-specific pathway libraries	Customized background for ORA	Return overlapping significant genes	Internal database availability and reproducibility	Web app source code availability	Asynchronous workflow
EnrichKit	Yes	Genomic coordinates annotation and aggregation, ORA, GSEA, Gene ID conversion	Yes	Enforced	Yes	Available (SQLite); reproducible	Yes, Github^a^ and Zenodo^b^	Yes
ShinyGO	Yes	ORA and complementary	No	Optional	No	Available (SQLite); not reproducible	Yes; Github^c^	No
Enrichr	No	ORA	No	Optional	No	Available (embedded links^d^); not reproducible	No	No
*g:Profiler*	Yes	ORA, ID conversion, orthologs search, SNP to gene	Yes	Optional	Yes	Partially available (APIs); not reproducible	No	No
MAGNET	No	ORA	No	Enforced	Yes	Available (embedded link for downloading GMT); reproducible	Yes, Github^e^	No
WebGestalt	Partial	ORA, GSEA, NTA	Yes	Optional	Yes	Partially available (APIs); reproducible	No	No

^a^ https://github.com/liulihe954/EnrichKitDB

^b^ https://doi.org/10.5281/zenodo.10257551

^c^ http://bioinformatics.sdstate.edu/data/current_version/convertIDs.db.tar.gz

^d^ https://maayanlab.cloud/Enrichr/#libraries

^e^ https://magnet-winterlab.herokuapp.com

### 4.1 Support for major livestock species

EnrichKit provides tailored support for each of the six species. EnrichKit is specifically designed to support six major livestock species where omics technologies are actively applied. These species include the cow (Bos taurus), goat (Capra hircus), sheep (Ovis aries), chicken (Gallus gallus), pig (Sus scrofa), and horse (Equus caballus). Other tools, such as ShinyGO and *g:Profiler*, also support these major livestock species. In contrast, WebGestalt 2019 supports a partial collection of these species. Additionally, other tools require converted gene IDs and do not offer species-specific options in the user interface.

### 4.2 Functionality

EnrichKit is designed to offer comprehensive analytical capabilities for the common outputs derived from omics technologies. This includes the biological interpretation of both coordinate-based and gene-list signals. Users have the flexibility to employ individual functions of EnrichKit for specific analyses or combine several functions for more in-depth investigations. For instance, one can aggregate a list of coordinates to generate gene lists of interest and subsequently run enrichment analyses on these lists to explore the underlying biological pathways. The tool *g:Profiler* also provides features to associate SNPs to gene, while other tools lack the focus of handing coordinate-based signals.

### 4.3 Availability of livestock-specific pathway libraries

EnrichKit efficiently compiles species-specific gene-set libraries for enrichment analysis, eliminating the need for cross-species gene ID conversion within its internal algorithms. This feature is instrumental in providing highly accurate and biologically relevant insights, particularly for non-human species. Similarly, tools like *g:Profiler* and WebGestalt 2019 also offer species-specific databases. However, other software that relies on common gene IDs as pathway component identifiers, despite having a broader range of libraries, may face challenges with information loss or mismatches during gene ID conversion. This issue often arises from the strategies that different providers use to match orthologs (Gabaldon and Koonin, 2013).

### 4.4 Support for customized background gene

EnrichKit enhances the accuracy and relevance of ORA by requiring users to provide a customized background gene set. This requirement significantly improves the reflection of the specific biological context of an experiment and mitigates bias towards well-studied genes. By enforcing this provision, EnrichKit ensures results that are not only more accurate but also biologically relevant and interpretable. Specifically, users must upload a comprehensive list of genes relevant to their study, distinguishing between significant and non-significant genes using an additional column. Tool MAGNET also offers this feature, while other tools may treat it as optional or lack it entirely.

### 4.5 Display of overlapping significant genes

EnrichKit can not only identify statistically significant gene sets but also delineate the specific genes that overlap between the input gene list and the candidate biological pathways or processes under investigation. This capability to pinpoint crucial driver genes enables users to gain deeper insights into the biological mechanisms observed in their data, paving the way for more targeted hypotheses for subsequent studies. Moreover, this feature aids in validating the relevance of the findings, ensuring that the results are not just statistically significant, but also biologically meaningful. Other tools, such as *g:Profiler*, MAGNET and WebGestalt 2019 also offer this feature, while other tools lack it.

### 4.6 Code availability and reproducibility

EnrichKit adopts an open-access approach to its internal database schema and web application source code, aligning with principles of transparency in scientific tools. This initiative allows users to reproduce the organizational structure of internal data items, e.g., gene features and candidate gene-set collections, and computational approaches, facilitating an informed understanding of the data sources and methodologies underpinning the software. This feature is intended to enhance user confidence in the outputs of EnrichKit, providing a foundation for accurate, rigorous, data-driven scientific inquiry. Specifically, EnrichKit, ShinyGO, Enrichr and MAGNET provide full access to all the gene-set collection reside in the software, while *g:Profiler* and WebGestalt 2019 provide partial access, i.e., APIs, to the internal database. In addition, EnrichKit, WebGestalt 2019 and MAGNET point to the first-order source data provider. Further, EnrichKit, ShinyGO and MAGNET provide access to the source code of the web application, which enhances transparency, allowing users to understand precisely how the application processes data, executes queries, and performs statistical analyses.

### 4.7 Asynchronous workflows

EnrichKit has implemented an asynchronous job handling system. This system permits users to submit tasks which are then executed in the background, allowing them to check the results at a later time upon completion; users are notified via email once the analysis is finished. Such workflows are designed to remove the necessity for users to stay online during prolonged processing times, significantly mitigating the inconvenience and risks linked to internet disconnections or session timeouts. Additionally, this methodology not only enhances server resource efficiency but also ensures the successful delivery of results, optimizing both user experience and system performance.

## 5 Conclusion

Here we introduce EnrichKit, a web-based platform that delivers robust solutions for multi-omics data interpretation in livestock genomics. It uniquely supports the annotation and functional characterization of genes across multiple livestock species, integrating the latest data from diverse public databases. EnrichKit stands out for its user-friendly design, facilitating access to advanced analytical tools for a broad spectrum of researchers. Overall, EnrichKit is poised to become a valuable resource in the field of livestock research, advancing the understanding of complex livestock genomics data.

## Data Availability

The original contributions presented in the study are included in the article/supplementary material, further inquiries can be directed to the corresponding authors.
